# Knowledge, attitudes, and practices regarding Crimean-Congo hemorrhagic fever in a high-prevalence suburban community, southeast of Iran

**DOI:** 10.1016/j.heliyon.2023.e23414

**Published:** 2023-12-09

**Authors:** Jalil Nejati, Mahdi Mohammadi, Hassan Okati-Aliabad

**Affiliations:** Health Promotion Research Center, Zahedan University of Medical Sciences, Zahedan, Iran

**Keywords:** Crimean-Congo hemorrhagic fever, Knowledge, Attitudes, Practice, Iran

## Abstract

Crimean Congo Hemorrhagic Fever (CCHF) is a zoonotic viral disease with a high mortality rate. The World Health Organization has classified it as a high-priority pathogen due to its severity. To manage this disease, knowledge, attitude, and practices (KAP) of the community play an important role. This study was conducted in a suburban area in southeastern Iran, an endemic region with a high occurrence of CCHF. A cross-sectional study was performed among 176 livestock farmers in Zahedan suburb, and data were collected through an interviewer-administered questionnaire. Single-factor and multi-factor analysis of variance was used to identify factors related to participants’ knowledge, attitude, and risk of CCHF. All statistical analyses were conducted using SPSS 24, and a significance level of p ≤ 0.05 was adopted to determine statistical significance. Overall, the knowledge level about CCHF was moderate. Participants demonstrated a better understanding of high-risk occupations compared to the transmission routes and symptoms of CCHF. Conversely, their knowledge about the prevention and treatment of CCHF was found to be inadequate. The participants exhibited a good attitude towards CCHF prevention; most considered it a dangerous disease, believed in the effectiveness of preventive measures, and demonstrated the ability to address perceived obstacles. High-frequency engagement in high-risk behaviors related to CCHF was observed among the study participants. In the multi-factor model, the most critical factors associated with knowledge were age (p = 0.044), CCHF infection (p = 0.047), and CCHF education (p = 0.004). Income (p = 0.001), keeping livestock at home (p = 0.028), and receiving CCHF education (p = 0.012) were the most critical factors associated with attitude toward the risk of CCHF. The most important factors related to CCHF high-risk behavior were age (p = 0.045), gender (p = 0.028), and handling livestock (p < 0.001). These findings emphasize the need for maintaining health educational efforts on CCHF.

## 1 Introduction

Crimean Congo Hemorrhagic Fever (CCHF) is a zoonotic viral disease with a mortality rate between 5 % and 50 % [1]. This tick-borne disease is prevalent in numerous regions including Africa, Asia, Eastern Europe, and the Middle East [2]. The evidence indicates a noticeable increase in cases of CCHF over the past decades. Both annual and periodic cases, as well as fatality rates, have shown an upward trend [3]. The seroprevalence of CCHF is 7.5-fold greater in at-risk individuals than in the general population. Farming/handling livestock, tick bites, and contact with secretions are recognized as prominent risk factors for CCHF seropositivity [4]. Some occupations such as butchers, slaughterhouse workers, physicians, medical staff, and health care workers are among high-risk groups due to more exposure to the vectors, infected livestock, or patients [5,6]. In the meantime, livestock farming increases the probability of human seropositivity about 6-fold [7].

Iran and its west and neighboring east countries, Turkey and Pakistan, are considered endemic countries for CCHF. This disease is now reported in 27 of 31 Iranian provinces [5,8,9]. A space-time analysis revealed the highest incidence of CCHF in Iran occurred in the eastern regions [10]. Sistan and Baluchestan Province, has the highest number of CCHF cases in the country. This province is still involved with malaria, and there have been reports of co-infection with both diseases [11]. The shared border with neighboring CCHF-endemic countries results in the illegal importation of livestock. It is estimated that approximately 300,000 livestock are illegally imported from Afghanistan into Iran each year [12].

The presence of CCHF seropositive livestock (cows, goats, and sheep) in Sistan and Baluchestan Province, as well as their high levels of importation, raises the hypothesis that infected livestock from neighboring countries might be contributing to this situation. However, it is important to note that there is currently insufficient evidence to definitively confirm this hypothesis [13,14]. About 75 % of the human cases have been reported in Zahedan County, the capital of Sistan and Baluchestan Province, with a high seroprevalence of CCHF among butchers and slaughterhouse workers [9,15]. Keeping livestock at home is a rural culture in the Zahedan suburb that can increase the chance of CCHF transmission [16].

The literature review shows that CCHF seroprevalence is higher due to livestock markets in suburban areas [17]. Nonetheless, public education about CCHF is generally performed for rural populations, and cities and their suburbs are less targeted [18]. In such circumstances, evaluating the knowledge, attitude, and practices (KAP) of the high-risk population can significantly contribute to the prevention and control of diseases in these hotspot zones. Most KAP studies toward CCHF in neighboring and other countries have included health care workers or physicians, and the general population are less questioned. A study conducted in 2013 in the two sub-counties of Kagadi district, Uganda, revealed that only 12.8 % of the respondents had knowledge about prevention measures against CCHF [19]. According to a cross-sectional survey conducted among the general population in Pakistan, only half of the participants had heard about CCHF infection. Among the study participants, 20.2 % demonstrated good knowledge, 33.3 % had positive attitudes, and 48.2 % showed good practices concerning CCHF [20]. In Pakistan, another KAP study conducted on herdsmen showed that 96 % of the respondents did not use any preventive measures against ticks due to a lack of knowledge [21]. A study conducted in Rafsanjan, Southern Iran, revealed that butchers generally had favorable attitudes and practice, but their knowledge regarding the subject was found to be at a moderate level [22]. In a study investigating rural Georgian villages, it was found that the majority (91.5 %) of participants were involved in high-risk activities associated with CCHF [23]. Surprisingly, only 29.7 % of the participants were aware of the potential risks of handling ticks without protection. Similarly, just 12.7 % of the respondents were aware of the risks associated with contact with animal blood. It was also noted that 28.8 % of the participants had knowledge of protective measures against CCHF and tick exposures; however, the implementation of these measures was relatively low, with only 54.3 % of participants employing them [23]. To prevent the spread of CCHF, it has been strongly recommended that personal protection measures and raising awareness among the population should be prioritized, especially due to the lack of a CCHF vaccine [24].

Considering traditional livestock farming and slaughtering at homes among residents of Zahedan outskirts, the present study was designed to investigate the knowledge, attitudes, and practices of households about CCHF in this high-risk area.

## 2 Materials and methods

### 2.1 Study design

A cross-sectional study was carried out from May to September 2021 to investigate the KAP of livestock farmers of Zahedan towards CCHF.

### 2.2 Study area

**Fig. 1:**
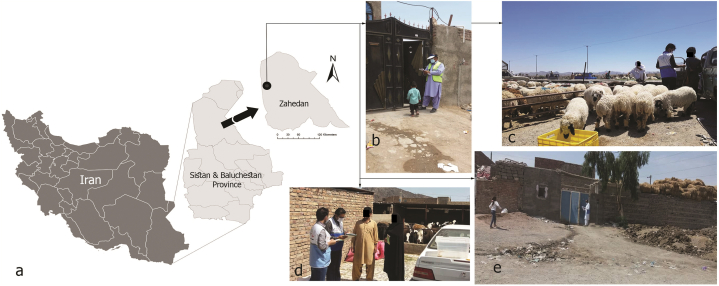
(A) Location of the study area "Shahrak-e− Gaavdaaran”, the suburb of Zahedan city. (b–e) photos show interviewers, (c,d) keeping livestock at home, (e) poor hygienic status in the alleys (Original photos taken by Jalil Nejati, 2021).

This study was conducted in Zahedan, the capital of Sistan and Baluchestan Province. It is located in southeastern Iran (60.86667° E and 29.49583° N) with a population of about 587,730 people. It has the most CCHF human cases in Iran as an endemic focus. This arid ecosystem, with dust storms, and a hot desert climate, has a high prevalence of the Hyalomma spp. ticks [25]. The current study was conducted in “Shahrak-e− Gaavdaaran” one of the suburbs of Zahedan. Previously, it was a livestock farming area, but now, it is a residential town. Despite an increasing number of residential areas [26], Zahedan continues to have a livestock owning population [16], which can increase the risk of CCHF transmission [[27], [28], [29]]. They live near their livestock and traditionally slaughter and butcher. This sociocultural condition led us to select this high CCHF burden suburb community for the current study (Fig. 1a–e).

### 2.3 Study participants and sampling

Participants were selected through a census method. The interviewers went door-to-door to visit every home in the study area and explained the purpose of the research to all adult members of livestock farmer households who met the inclusion criteria. These criteria included a willingness to participate in the survey, residency in Shahrak-e− Gaavdaaran, Zahedan livestock town, for at least six months, and being over 18 years of age. We excluded individuals with severe physical or mental disabilities who were unable to participate and those who showed symptoms of CCHF during the survey to avoid potential confusion in the study results caused by them gaining additional information.

### 2.4 Data collection method

Data were collected through an interviewer-administered questionnaire. Before conducting the data collection process, six interviewers who were local health workers with the ability to speak and understand Persian and Balochi languages received training in the health center of the study area (Shahrak-e-Gaavdaaran). The training covered the purpose of the study, explanations of the various questionnaire sections, communication with participants, data collection procedures, and obtaining informed and confidential consent. The questionnaire utilized by the interviewers was developed based on expanded relevant articles and the national (Persian) guide for CCHF prevention and control [30]. It included questions related to the participants' socio-demographic characteristics, such as age, gender, marital status, income, education, and job, as well as their history of CCHF infection, tick bites, exposure to patients, handling and slaughtering of livestock, and the keeping of livestock at home. Furthermore, the questionnaire also included questions to assess the participants’ knowledge, attitudes, and practices regarding CCHF (see supplementary file S1).

Knowledge about CCHF was evaluated through 31 questions that covered topics such as way of transmission, symptom, high-risk occupations, prevention, and treatment. The questions were answered on a false/true/do not know basis. The correct answers were assigned 1 point, and the incorrect answers were given zero points. The total knowledge score ranged from 0 to 31.

Attitude towards CCHF was assessed with 12 questions, including the vulnerability of individuals dealing with livestock, severity of CCHF, perceived barriers to preventive measures, and efficacy. All answers were based on a 3-point Likert scale ranging from disagree (1) to agree (3). The total Attitude score ranged from 12 to 36.

The questionnaire included nine questions for assessing participants' CCHF prevention practices. Based on the participants' answers to the questions concerning their practices, the risk score was calculated. Two questions were about the use of protective tools (hat, mask, butcher robe, plastic butcher apron, boots, and gloves) while handling and slaughtering livestock. A score of 1 was assigned if the tool was not used and 0 if it was used. There was also a yes/no question about slaughtering, with a score of 1 assigned if the person had slaughtered livestock. There were 5 questions with a 3-point Likert scale scored 1 for ‘always’, 0.5 for ‘sometimes’, and 0 for ‘never’. used the following scores: 1 for ‘nearly every day’, 0.8 for ‘once a week’, 0.6 for ‘once a month’, 0.4 for ‘once a year’, and 0 for ‘never’.

In this study, we classified the total scores for knowledge, attitude, and practice based on Bloom's taxonomy. A score exceeding 80 % was considered ‘good’, while those ranging from 60 % to 80 % were categorized as ‘moderate’, and scores below 60 % were classified as ‘poor’ [31].

To ensure clarity, relevance, and consistency, we conducted a pilot study involving 30 participants who were subsequently excluded from the final sample. The face and content validity of the questionnaire (assessed through the content validity index and content validity ratio) were confirmed by a panel of 10 experts in health education, medical entomology, infectious diseases, and epidemiology at Zahedan University of Medical Sciences. Cronbach's alpha was more significant than 0.70, indicating acceptable internal consistency.

### 2.5 Data analysis

Qualitative and quantitative variables were described as frequency (percent) and mean ± standard deviation (SD). Single-factor and multi-factor analysis of variance was used to identify factors related to participants’ knowledge, attitude, and risk of CCHF. The significant variables identified in the single-factor model were entered into the multi-factor model. All statistical analyses were performed using SPSS 24 for Windows [SPSS, Chicago, IL, USA], and p-value of ≤0.05 was considered statistically significant.

## 3 Results

### 3.1 Demographic analysis

A total of 176 people with a mean age of 37.82 ± 14.24 participated in this study. The majority of participants were male (n = 107; 60.8 %) and married (n = 154; 88 %) with an income of less than 50 million IRR (n = 106; 62.4 %). Approximately, one-third of individuals (n = 60; 37.3 %) were illiterate, and 52 % (n = 91) held an elementary or secondary school degree. Most people lived with a spouse and children (n = 149; 84.7 %). About 40 % (n = 68) of them were livestock farmers, and 55.7 % (n = 98) had a specific place for keeping livestock at home. Participants stated histories of handling livestock (n = 119; 67.6 %), slaughtering livestock (n = 57; 32.6 %), exposure to CCHF patients (n = 1; 6.3 %), tick bite 6.3 % (n = 11), CCHF infection (n = 7; 4 %), and receiving education related to CCHF (n = 77; 43.8 %).

### 3.2 Knowledge analysis

The total mean knowledge score was 23.60 ± 3.96 out of 36. Specifically, the mean knowledge regarding ways of CCHF transmission was 7.89 ± 2.52 out of 12. The majority (n = 140; 79.5 %) of individuals demonstrated good knowledge about CCHF transmission ways. Nevertheless, less than half of the participants expressed that drinking unboiled milk (n = 42; 23.9 %), having social contact with CCHF patients (n = 75; 42.6 %), inhaling air (n = 81; 46 %), and drinking contaminated water (n = 74; 42 %) are not ways of CCHF transmission.

The average knowledge score regarding CCHF symptoms was 7.12 ± 2.65 out of 9. Bleeding, fever, headache, and muscular pain were reported by more than 85 % of participants as symptoms of CCHF. Joint pain, diarrhea, nausea, and vomiting were mentioned by about 75 % of them. Furthermore, around 69 % of individuals reported weakness and bruising.

The mean of knowledge about high-risk occupations was 4.01 ± 1.10 out of 5. More than 88 % of participants knew that livestock farmers (n = 164; 93.2 %), butchers (n = 162; 92 %), and slaughterhouse workers (n = 155; 88.1 %) are at high risk of CCHF infection, and 76 % (n = 134) recognized that vets are at risk of the disease. In addition, half of the participants (n = 90; 51.1 %) did not consider farming as a high-risk job.

The mean knowledge score regarding the prevention and treatment of CCHF was 2.09 ± 1.50 out of 5. Most people were unaware that infected livestock can be asymptomatic (n = 97; 56 %), that medicine may not always be effective in curing CCHF patients (145 = 83.4 %), and that a vaccine is not available for CCHF (n = 113; 64.9 %). However, over half of participants correctly stated that CCHF incidence is not most prevalent during cold seasons (n = 9; 52.3 %), and that consuming the meat of livestock immediately after slaughtering should be avoided (n = 107; 61.5 %) (Table 1).

**Table 1:** Frequency distribution of Knowledge items about CCHF.

Category\ Knowledge variables	Correct answer n (%)
**Ways of transmission**	
Tick bite	150 (85.2)
Crushing tick with hand	142 (80.7)
Contact with CCHF patient blood	140 (79.5)
Contact with CCHF patient stool, urine, or saliva	108 (61.4)
Contact with CCHF-infected livestock fluid	133 (75.6)
Contact with CCHF infected carcasses	146 (83.0)
Contact with CCHF-infected livestock blood	150 (85.2)
Eating uncooked meat of CCHF-infected livestock	147 (83.5)
Drinking unboiled milk of CCHF-infected livestock	42 (23.9)
Social contact (shaking hands, kissing, …) with CCHF patient	75 (42.6)
Air inhalation	81 (46.0)
Drinking contaminated water	74 (42.0)
**Symptoms of CCHF**	
Bleeding	150 (85.2)
Fever	159 (90.3)
Headache	153 (86.9)
Muscular pain	150 (85.2)
Joints pain	136 (77.3)
Nausea & Vomiting	133 (75.6)
Diarrhea	129 (73.3)
Weakness	120 (68.6)
Bruising	121 (69.1)
**Jobs at risk**	
Livestock farmers	164 (93.2)
Vets	134 (76.1)
Butchers	162 (92.0)
Slaughterhouse workers	155 (88.1)
Farmers	90 (51.1)
**Prevention and treatment**	
Infected livestock is often symptomatic	76 (43.9)
CCHF is curable with medicine	29 (16.7)
Eating meat just after slaughtering	107 (61.5)
CCHF is mostly prevalent in cold seasons	91 (52.3)
CCHF vaccine is available	61 (34.7)

### 3.3 Attitude analysis

The mean total attitude score toward CCHF was 29.48 ± 6.36 out of 36. Most participants believed they could reduce their risk of contracting CCHF by avoiding handling livestock (n = 14; 81 %), using safety equipment while slaughtering (n = 122; 70.5 %), and not relying on livestock for their health (n = 114; 65.9 %). Furthermore, they perceived CCHF as a dangerous disease (118 = 67.8 %) that cannot be prevented by physical strength alone (n = 121; 69.5 %). Participants also emphasized the importance of using safety equipment during both slaughtering (n = 102; 59 %) and when handling livestock (n = 102; 58.6 %), even if it caused discomfort (Table 2).

**Table 2:** Frequency distribution of attitude items towards CCHF.

Attitude variables	Agree n (%)	Neutral n (%)	Disagree n (%)
People who expose to livestock are more at risk of CCHF	141 (81.0)	18 (10.3)	15 (8.6)
I will not get CCHF because my body is strong	22 (12.6)	31 (17.8)	121 (69.5)
CCHF is not a dangerous disease	29 (16.7)	27 (15.5)	118 (67.8)
My livestock is healthy and I do not need to wear safety equipment (like gloves, boots, etc)	28 (16.2)	31 (17.9)	114 (65.9)
There is no need for safety equipment (like gloves, boots, etc)when slaughtering	22 (12.7)	29 (16.8)	122 (70.5)
Using safety equipment (like gloves, boots, etc) while handling livestock is suffering	36 (20.7)	36 (20.7)	102 (58.6)
Using safety equipment (like gloves, boots, etc) while handling livestock is not effective for CCHF prevention	27 (15.7)	35 (20.3)	110 (62.5)
Using safety equipment (like gloves, boots, etc) while slaughtering is not effective for CCHF prevention	25 (14.5)	32 (18.5)	116 (67.1)
Using safety equipment (like gloves, boots, etc) while slaughtering is suffering	37 (21.4)	34 (19.7)	102 (59.0)
I cannot afford to buy safety equipment like gloves, boots, etc	43 (24.7)	34 (19.5)	97 (55.7)
I forget to use safety equipment (like gloves, boots, etc) when slaughtering or handling livestock	59 (34.1)	30 (17.3)	84 (48.6)
Spraying livestock and their keeping places is not effective in preventing CCHF	32 (18.4)	26 (14.9)	116 (66.7)

The frequency analysis includes a maximum of 4 (2.2 %) missing.

### 3.4 Practice (high-risk behavior) analysis

The mean score for CCHF-related high-risk behavior was 12.05 ± 1.79 out of 18. More than two-thirds of the participants reported not wearing protective tools (such as a hat, mask, boot, butcher's robe, and plastic apron) while handling livestock or slaughtering. In the mentioned conditions, wearing gloves was reported by 40.8 % (n = 40) and 46.9 % (n = 30) of participants, respectively (Table 3). Approximately half of the participants 50.9 % (n = 85) admitted to not using gloves while chopping the slaughtered meat, and 37.8 % (n = 65) consumed the meat immediately after slaughtering. (Table 4).

**Table 3:** Frequency distribution of high-risk behaviors towards CCHF disease while handling and slaughtering livestock.

Do not wear a	When handling livestock (n = 98)	When slaughtering livestock (n = 64)
Hat	95 (96.9)	61 (95.3)
Mask	77 (78.6)	47 (73.4)
Butcher's robe	84 (85.7)	52 (81.2)
Butcher's plastic apron	76 (77.6)	49 (76.9)
Boot	67 (68.4)	46 (71.9)
Gloves	40 (40.8)	30 (46.9)

The frequency analysis includes a maximum of 4 (2.2 %) missing.

**Table 4:** Frequency distribution of high-risk behaviors towards CCHF disease after slaughtering livestock.

Practice variables	Always n (%)	Often n (%)	Never n (%)
Eating a slice of the liver while slaughtering	1 (0.6)	5 (2.9)	166 (96.5)
Consuming the meat just after slaughtering livestock	27 (15.7)	38 (22.1)	107 (62.2)
Chopping the slaughtered livestock meat without wearing gloves	27 (16.2)	55 (32.9)	85 (50.9)

a The frequency analysis includes a maximum of 6 (3.4 %) missing.

### 3.5 One and multi-factor model analysis

Knowledge was significantly lower among young people compared to elderly (p = 0.01). Conversely, livestock farmers (p = 0.019), individuals with a history of CCHF infection (p = 0.026) or handling livestock (p = 0.012), and those who received education about CCHF disease (p < 0.001) demonstrated significantly higher levels of knowledge (Table 5). The multi-factor model indicated that age (p = 0.044), CCHF infection (p = 0.047), and CCHF education (p = 0.004) were the most important factors associated with knowledge.

**Table 5:** Factors associated with knowledge, attitude, and risk of CCHF in a one-factor model.

Variable\ Category	Knowledge		Attitude		Risk	
	Mean ± SD	p-value	Mean ± SD	p-value	Mean ± SD	p-value
**Age (year)**						
18–25	22.13 ± 3.87	0.010	29.17 ± 5.87	0.460	11.15 ± 2.48	**0.028**
26–35	23.25 ± 4.40	0.082	29.71 ± 5.62	0.216	12.25 ± 1.49	0.865
36–55	24.27 ± 3.38	0.578	30.24 ± 6.02	0.126	12.26 ± 1.57	0.859
More than 55	24.78 ± 3.50	Ref.	27.93 ± 8.77	Ref.	12.18 ± 1.76	Ref.
**Gender**						
Female	22.94 ± 4.50	0.076	29.68 ± 5.68	0.749	11.60 ± 2.09	**0.007**
Male	24.03 ± 3.53	Ref.	29.36 ± 6.79	Ref.	12.34 ± 1.51	Ref.
**Marital status**						
Single	23.28 ± 3.86	0.713	30.11 ± 4.84	0.637	11.67 ± 1.83	0.345
Married/Widow	23.64 ± 3.99	Ref.	29.44 ± 6.53	Ref.	12.09 ± 1.79	Ref.
**Income (million IRR)**						
Less than 50	23.28 ± 4.24	0.501	30.19 ± 5.66	<0.001	11.72 ± 1.84	0.068
50–100	24.09 ± 3.43	0.215	29.15 ± 6.96	0.002	12.53 ± 1.32	0.657
>100	22.40 ± 3.37	Ref.	22.60 ± 6.74	Ref.	12.80 ± 2.97	Ref.
**Education**						
Illiterate	22.72 ± 4.70	0.438	28.35 ± 6.82	0.378	12.15 ± 1.51	0.070
Elementary school	23.80 ± 3.04	0.958	30.70 ± 5.84	0.069	11.85 ± 1.77	0.**027**
Secondary school	24.38 ± 3.32	0.738	30.23 ± 5.64	0.101	12.09 ± 2.10	0.061
High school diploma	23.81 ± 4.76	0.971	29.47 ± 5.79	0.247	11.62 ± 1.67	**0.025**
University graduates	23.88 ± 4.05	Ref.	26.25 ± 9.25	Ref.	13.38 ± 1.94	Ref.
**Job**						
Livestock farmers	24.41 ± 2.87	0.019	28.16 ± 7.77	0.032	12.46 ± 1.91	0.**018**
Others	22.98 ± 4.48	Ref.	30.28 ± 5.10	Ref.	11.79 ± 1.68	Ref.
**History of CCHF infection**						
Yes	26.86 ± 2.34	0.026	32.28 ± 6.13	0.235	12.21 ± 1.25	0.811
No	23.47 ± 3.96	Ref.	29.36 ± 6.36	Ref.	12.05 ± 1.81	Ref.
**History of handling livestock**						
Yes	24.12 ± 3.79	0.012	29.68 ± 6.39	0.552	12.46 ± 1.56	**<0.001**
No	22.53 ± 4.12	Ref.	29.07 ± 6.33	Ref.	11.23 ± 1.96	Ref.
**History of slaughtering livestock**						
Yes	23.56 ± 4.08	0.929	27.50 ± 7.51	0.004	12.85 ± 1.61	**<0.001**
No	23.62 ± 3.94	Ref.	30.44 ± 5.54	Ref.	11.68 ± 1.76	Ref.
**History of exposure to CCHF patient**						
Yes	25.36 ± 4.30	0.128	33.18 ± 3.94	0.046	12.54 ± 0.93	0.350
No	23.48 ± 3.92	Ref.	29.23 ± 6.42	Ref.	12.02 ± 1.8	Ref.
**History of tick bite**						
Yes	24.18 ± 3.46	0.618	29.18 ± 7.10	0.872	13.32 ± 1.42	**0.015**
No	23.56 ± 4.00	Ref.	29.50 ± 6.33	Ref.	11.97 ± 1.79	Ref.
**A specific place for keeping livestock at home**						
Yes	23.63 ± 3.86	0.910	28.72 ± 6.75	0.079	12.34 ± 1.54	**0.016**
No	23.56 ± 4.11	Ref.	30.42 ± 5.75	Ref.	11.69 ± 2.02	Ref.
**Received training on CCHF**						
Yes	24.79 ± 3.35	<0.001	30.72 ± 6.27	0.023	12.19 ± 1.52	0.379
No	22.68 ± 4.16	Ref.	28.52 ± 6.30	Ref.	11.95 ± 1.98	Ref.

The frequency analysis includes a maximum of 4 (2.2 %) missing.

In contrast to livestock farmers (p = 0.032) and individuals with a history of slaughtering livestock (p = 0.004), those with an income below 100,000,000 IRR (p = 0.002), a history of exposure to CCHF patient (p = 0.046), and people who received education about CCHF (p = 0.023) had significantly better attitudes toward the risk of CCHF (Table 5). Income (p = 0.001), keeping livestock at home (p = 0.028), and receiving CCHF education (p = 0.012) were identified as the most important factors associated with attitude towards the risk of CCHF, according to the multi-factor model.

CCHF-related high-risk behavior was significantly less in young people (p = 0.028), females (p = 0.007), and people with an elementary (p = 0.027) or high school education degree (p = 0.025) compared to university graduates. Conversely, high-risk behavior was greater in livestock farmers (p = 0.018), individuals who had a specific place for keeping livestock at home (p = 0.016), those with a history of handling livestock (p < 0.001), slaughtering livestock (p < 0.001), or a tick bite (p = 0.015) (Table 5). According to the multi-factor model, age (p = 0.045), gender (p = 0.028), and handling livestock (p < 0.001) were the most important factors associated with CCHF-related high-risk behavior.

## 4 Discussion

This study aimed to assess the knowledge, attitudes, and practices regarding CCHF among livestock farmers residing on the outskirts of Zahedan. Overall, the knowledge level among Zahedan's livestock farmers about CCHF was moderate. Participants demonstrated a better understanding of high-risk occupations compared to the transmission routes and symptoms of CCHF. Conversely, their knowledge about the prevention and treatment of CCHF was found to be inadequate. However, the participants exhibited a good attitude towards CCHF prevention; the majority of them considered it a dangerous disease, believed in the effectiveness of preventive measures, and demonstrated the ability to address perceived obstacles. High-frequency engagement in high-risk behaviors related to CCHF was observed among the study participants. The most important factors related to knowledge were age, CCHF infection, and education about CCHF. The pivotal factors influencing attitude toward the risk of CCHF were income, keeping livestock at home, and receiving a CCHF education. Age, gender, and handling livestock emerged as the primary contributors to high-risk behavior associated with CCHF.

Due to the high fatality rate of CCHF, it has been classified as a high-priority pathogen by the World Health Organization [32]. This study was conducted in a county with the most CCHF cases, a province with the largest high-risk area for occurrence, and a country that still has the highest incidence of CCHF in the world [15,25,33,34]. Although some studies were found on healthcare professionals and butchers’ KAP in Iran [35,36], it is the first KAP study on Iranian livestock farmers in a high CCHF burden community. The current study showed that the average total knowledge and the behavior score were not acceptable. In this situation, the One-Health approach that encompasses biosurveillance of ticks, animals, and humans is essential to monitor and prevent outbreaks in humans effectively [37].

Through our survey, it has been known that more than half of interviewees kept livestock at home, one-third were illiterate, and more than half of them had low incomes. Similarly, several studies in the eastern neighbor, Pakistan, showed a high percentage of owners that kept livestock at home. A low literacy rate or illiteracy with low income among herdsmen has also been documented in some studies conducted in that country, especially in Balochistan Province, with the highest prevalence of CCHF [21,38,39]. It can be concluded that lower income, along with a lack of education and awareness, affects the prevention of CCHF by reducing the appropriate conditions for keeping livestock and the motivation to purchase personal protective tools. A KAP study conducted in the western neighbor, Turkey, an endemic country with a high prevalence of CCHF, found that 81 % of illiterate people and even 72 % of individuals with elementary education levels had never heard anything about CCHF [18]. Another study in Turkey on risk factors of CCHF showed that the most seropositivity (Anti-CCHFV IgG) had been detected in low-educated and low-income groups [8]. These results show the importance of education and economic conditions, especially for people dealing with livestock husbandry.

The current study showed that the average total knowledge score was not ideal (23.60 ± 3.96 out of 36). More than half of the participants held an elementary or secondary school degree. In a survey conducted on the general population in Ankara, Turkey, the average knowledge score was lower than the median. In that study, only 37 % of people had an education level of high school or higher. The researchers found statistically significant relationships between knowledge scores and education, suggesting that individuals with a high school education or higher will have more knowledge about CCHF [18]. However, in our investigation, CCHF-related high-risk behavior was significantly less in people with an elementary or high school education than university graduates. Older age and university education did not prevent risky behavior. It seems that a higher level of literacy does not necessarily mean more knowledge about the methods of transmission and prevention of CCHF. For example, a study conducted on medical students in Iran showed that they did not receive any training on standard isolation precautions [35]. Similarly, a KAP study among butchers in Libya showed no relationship between awareness and the level of education, and even their practices in some questions [40]. In Uganda, a study revealed that there were low levels of knowledge and awareness regarding CCHF among the population. As a result, they strongly recommended the implementation of educational programs aimed at increasing awareness of CCHF in communities and promoting preventive measures for the disease [19]. It can indicate the necessity of practical and assessable training for all age groups and educational levels.

Despite the poor total knowledge of CCHF, our study showed that the mean knowledge about its symptoms was good. Similarly, in Turkey, respondents knew the transmission ways of CCHF and mentioned fever as the most common symptom [18]. Contrarily, an investigation conducted in the neighboring Pakistani province of Balochistan revealed that none of the respondents were aware of the incubation period, transmission methods, or symptoms of CCHF. The study indicated that the familiarity of rural communities with CCHF symptoms is closely related to the incidence of patients in the local community [41].

In our study, the knowledge of symptoms in young people was significantly less than elderly, and it was higher in livestock farmers and people with a history of CCHF infection. This result can be related to the possibility of patient observation or experiencing the disease by the responder. Our findings showed that individuals with a history of exposure to or visits with CCHF patients had significantly good attitudes towards the risk of CCHF. Undoubtedly, the population's knowledge can be valuable for early diagnosis and treatment, especially throughout the seasonal peak of CCHF [18].

A study conducted in Pakistan revealed a poor attitude among the rural population [39] However, in our study, the mean total attitude score for CCHF was 29.48 out of 36, which, according to Bloom's taxonomy, was considered good (above 80 % of the total attitude score) [31]. Unlike the present study, in which most participants believed avoiding exposure to livestock and using safety equipment while slaughtering would reduce the risk of CCHF, in the Pakistani province of Baluchistan, all respondents answered that they could not do anything against CCHF and it is all in the hands of God [41]. So, people's culture and religious beliefs should be considered in the education of CCHF.

In the present study, the practice score was not good according to the Bloom's taxonomy, which could be a contributing factor to the occurrence of CCHF outbreaks in the study area. The majority of residents did not use protective equipment when handling or slaughtering livestock. Additionally, approximately half of the participants did not wear gloves while chopping the meat. An investigation of health workers in the study area showed that only 44 % wore gloves and masks when exposed to CCHF patients, and 22 % did not observe any safety measures [35], which supports our findings. Failure to implement knowledge into practice can be a socio-cultural issue that plays an important role in CCHF outbreaks [42].

### 4.1 Limitations

One limitation inherent to this study is its utilization of a cross-sectional design and data collection approach. Therefore, the results of the study should be interpreted with caution. The cross-sectional design restricts our ability to establish causal relationships. Additionally, the study places significant reliance on self-reported data. This dependence on self-reporting could potentially introduce recall or social desirability bias, thereby influencing the precision of participants' understanding, attitudes, and behaviors about Crimean-Congo hemorrhagic fever. Furthermore, the study faced a potential limitation when using the Likert scale to measure attitudes. Scoring for neutral (neither agree, nor disagree) on the attitude scale, meant they may have met the threshold for a ‘moderate’ attitude when expressing neutral. However, a simple three-point Likert scale was necessary due to concerns about participant illiteracy or low education levels. It seems the probability of everyone choosing the neutral option is very low. Nonetheless, the authors reviewed the data to investigate this issue and found no such cases. The findings of this study offer valuable insights for policymakers and healthcare administrators, aiding in formulating preventive strategies for CCHF, particularly within this vulnerable population.

## 5 Conclusion

The study revealed that Zahedan's livestock farmers had moderate knowledge regarding CCHF, but they held a good attitude towards the disease. Furthermore, the study highlighted a significant prevalence of high-risk behaviors associated with CCHF among the participants. These findings emphasize the need for continuous health education programs that focus on CCHF in endemic areas. By addressing these knowledge gaps and promoting positive attitudes, we can potentially reduce the risks posed by this formidable disease.

## Ethics statement

The study protocol was reviewed and approved by the Ethical Review Committee of Zahedan University of Medical Sciences (IR.ZAUMS.REC.1400.013). Written informed consent was obtained from all study participants. Additionally, the participants and interviewers provided written consent for their photos to be included in the publication. The study was conducted in accordance with the Declaration of Helsinki.

## Funding statement

This study was supported by the 10.13039/501100004847Zahedan University of Medical Sciences.

## Data availability statement

Data will be made available on request.

## CRediT authorship contribution statement

**Jalil Nejati:** Writing – original draft, Investigation, Conceptualization. **Mahdi Mohammadi:** Formal analysis, Data curation. **Hassan Okati-Aliabad:** Writing – review & editing, Methodology.

## Declaration of competing interest

The authors declare that they have no known competing financial interests or personal relationships that could have appeared to influence the work reported in this paper.
