# Supplementary material for: Knowledge, attitudes, and practices regarding Crimean-Congo hemorrhagic fever in a high-prevalence suburban community, southeast of Iran
Source: Heliyon. 2023 Dec 9;10(1):e23414. doi: 10.1016/j.heliyon.2023.e23414 (PMC10750147; doi:10.1016/j.heliyon.2023.e23414)
Supplement: Multimedia component 2 [file mmc2.pdf]

Zahedan University Of Medical Sciences

Research Ethics Certificate

Approval ID:	IR.ZAUMS.REC.1400.013	Approval Date:	2021-04-04
Evaluated by:	Zahedan University Of Medical Sciences		
Status:	Approved		
Approval Statement:	The project was found to be in accordance to the ethical principles and the national norms and standards for conducting Medical Research in Iran.<br>Notice:<br>1. Although the proposal has been approved by the research ethics committee, meeting the professional and legal requirements is the sole responsibility of the PI and other project collaborators.<br>2. This certificate is reliant on the proposal/documents received by this committee on 2021-04-04. The committee must be notified by the PI as soon as the proposal/documents are modified.		
Proposal Title:	knowledge, attitude, and practice related to Crimean Congo Hemorrhagic Fever (CCHF) among some suburban residents of Zahedan		
Principal Investigator:	Name: Jalil Nejati<br>Email: jalilnejati@yahoo.com		

Dr. Seyed Mohammad Hashemi Shahri
Director of University/Regional Research Ethics Committee
Zahedan University Of Medical Sciences

Dr. Noor Mohammad Bakhshani
Secretary of University/Regional Research Ethics Committee
Zahedan University Of Medical Sciences
