# Supplementary material for: Knowledge, attitudes, and practices regarding Crimean-Congo hemorrhagic fever in a high-prevalence suburban community, southeast of Iran
Source: Heliyon. 2023 Dec 9;10(1):e23414. doi: 10.1016/j.heliyon.2023.e23414 (PMC10750147; doi:10.1016/j.heliyon.2023.e23414)
Supplement: Multimedia component 3 [file mmc3.pdf]

Questionnaire code:

Dear fellow-citizen

With your cooperation and valuable answers to this questionnaire, the research entitle “knowledge, attitude, and practice related to Crimean Congo Hemorrhagic Fever (CCHF) among some suburban residents of Zahedan” will be conducted. Therefore, please read it and answer the questions carefully (3 pages). If you have any doubts about the questions, you can ask for guidance from the expert who gave it.

It should be noted that all answers will be confidential and used to prevent CCHF.

	**Demographic Information**	
1	Age	.... years
2	Gender	1- Female☐ 2- Male☐
3	Marital status	1- Single☐ 2- Married☐ 3- Divorced☐ 4- Dead Spouse/Widow☐
4	Living situation	1- Living with wife and children☐ 2- Living with wife☐ 3- Living with children☐ 4- Living with parents ☐ 5- I live alone☐
5	Your health status from your own point of view	1- Very bad☐ 2- Bad☐ 3- Average☐ 4- Good☐ 5- Very good☐
6	Amount of income	1- Less than 50 million IRR☐ 2- Between 50-100 million IRR☐ 3- More than 100 million IRR☐
7	Level of education	1- Illiterate☐ 2- Elementary ☐ 3- Middle school☐ 4- Diploma☐ 5- University☐
8	Occupation	1- livestock farmers☐ 2- Butcher☐ 3- Slaughterhouse worker☐ 4- Farmer☐ 5- Other items: please mention ....
9	History of CCHF infection	1-Yes☐ 2- No☐
10	History of handling livestock	1-Yes☐ 2- No☐
11	History of slaughtering livestock	1-Yes☐ 2- No☐
12	History of exposure with CCHF patient	1-Yes☐ 2- No☐
13	Presence a specific place for keeping livestock in the home	1-Yes☐ 2- No☐
14	History of tick bite	1-Yes☐ 2- No☐
15	History of receiving education about CCHF	1-Yes☐ 2- No☐

Please determine the way of CCHF transmission from the following options.		Yes	No	I don't know
1	Tick bite			
2	Crushing ticks by hand			
3	Direct contact with the blood of CCHF patient			
4	Contact with feces, urine and saliva of CCHF patient			
5	Direct contact with body fluids of infected livestock			
6	Direct contact with the carcass of an infected livestock			
7	Direct contact with the blood of infected livestock			
8	Eating uncooked meat of infected livestock			
9	Eating unboiled milk of infected livestock			
10	Social contacts such as shaking hands and kissing infected patients			
11	Air inhalation			
12	Contaminated water			
**Please determine which of the following is CCHF symptom/s.**		**Yes**	**No**	**I don't know**
1	Bleeding			
2	Fever			
3	Headache			
4	Muscle pain			
5	Joint's pain			
6	Nausea and vomiting			
7	Diarrhea			
8	Weakness			
9	Bruise			
**Please determine the group/s most at risk of CCHF.**		**Yes**	**No**	**I don't know**
1	Herdsman			
2	Veterinarians			
3	Butchers			
4	Slaughterhouse Workers			
5	Farmers			
**Please answer the following questions:**		**Yes**	**No**	**I don't know**
1	Infected livestock with CCHF is symptomatic mostly			
2	CCHF is completely treatable with medicine.			
3	Livestock meat can be used immediately after slaughter.			
4	CCHF is more common in the cold seasons.			
5	CCHF vaccine is available in drug stores.			

Please answer the following questions that are related to your attitude towards the CCHF.		Agree	Neutral (neither agree nor disagree)	Disagree
1	In my opinion, people who expose with livestock are more at risk of CCHF			
2	Because my body is strong, I will not get CCHF			
3	In my opinion, CCHF is not a dangerous disease			
4	In my opinion, my livestock are healthy and I do not need to wear safety equipment such as gloves, boots, etc. when handling them.			
5	In my opinion, I do not need to use safety equipment such as gloves, boots, etc. during slaughtering livestock.			
6	In my opinion, using safety equipment such as gloves, boots, etc. while handling livestock is annoying.			
7	In my opinion, using safety equipment (like gloves, boots, etc.) while handling livestock is not effective for CCHF prevention.			

8	In my opinion, using safety equipment such as gloves, boots, etc. during livestock slaughtering is not effective in CCHF prevention.			
9	In my opinion, using safety equipment such as gloves, boots, etc. during livestock slaughtering is annoying.			
10	I cannot afford to buy safety equipment like gloves, boots, etc.			
11	I forget to use safety equipment such as gloves, boots, etc. while slaughtering or handling livestock.			
12	In my opinion, spraying livestock shelters is not effective in CCHF prevention.			

**Please answer the following questions that are related to your practice towards the CCHF.**		
1	Do you slaughter livestock yourself?	1- Yes☐ 2- No☐
2	How often do you slaughter livestock?	1- Almost every day☐ 2- Once a week☐ 3- Once a month☐ 4- Once a year☐ 5- I do not slaughter livestock☐
3	Which of the following safety tools do you use during slaughtering livestock? It is possible to choose more than one item.	1- Hat☐ 2- Mask☐ 3- Butcher's robe☐ 4- Plastic apron☐ 5- Boots☐ 6-Gloves☐ 4- I do not slaughter livestock☐
4	Do you have a habit of keeping the knife between your lips during slaughtering livestock?	1- Always☐ 2- Sometimes☐ 3- Never☐ 4- I do not slaughter livestock☐
5	Do you have a habit of eating a piece of livestock raw liver during slaughtering?	1- Always☐ 2- Sometimes☐ 3- Never☐
6	Do you have a habit of consuming the meat immediately after slaughtering?	1- Always☐ 2- Sometimes☐ 3- Never☐
7	Which of the following safety equipment/s do you wear when handling livestock? It is possible to choose more than one item.	1- Hat☐ 2- Mask☐ 3- Butcher's robe☐ 4- Plastic apron☐ 5- Boots☐ 6- loves☐ 4- I do not slaughter livestock☐
8	Do you use gloves when chopping the slaughtered meat?	1- Always☐ 2- Sometimes☐ 3- Never☐
9	Do you spray the place where your livestock are kept?	1- Always☐ 2- Sometimes☐ 3- Never☐

END.

Thanks for your participation
